# The pressure exerted on the tracheal wall by two endotracheal tube cuffs: A prospective observational bench-top, clinical and radiological study

**DOI:** 10.1186/1471-2253-10-21

**Published:** 2010-12-09

**Authors:** Alex Doyle, Ramai Santhirapala, Martin Crowe, Mark Blunt, Peter Young

**Affiliations:** 1 Critical Care Trainee, Department of Anaesthesia and Critical Care, Queen Elizabeth Hospital, Kings Lynn, Norfolk, UK, PE30 4ET; 2 Consultant in Radiology, Department of Anaesthesia and Critical Care, Queen Elizabeth Hospital, Kings Lynn, Norfolk, UK, PE30 4ET; 3 Consultant in Anaesthesia and Critical Care, Department of Anaesthesia and Critical Care, Queen Elizabeth Hospital, Kings Lynn, Norfolk, UK, PE30 4ET

## Abstract

**Background:**

The Lotrach endotracheal tube has a unique low-volume, low-pressure (LVLP) cuff, which has been designed to prevent pressure injury to the tracheal wall. We aimed to estimate the pressure exerted on the tracheal wall by the LVLP cuff and a conventional cuff in a bench-top, clinical and radiological study.

**Method:**

In the bench-top study, a model trachea was intubated with the LVLP cuff and the conventional cuff. The cuff pressure was controlled using a constant pressure device. We assessed the pressure exerted on the tracheal wall by measuring the ability of the cuffs to support a column of water using a standard protocol. In the clinical study, we tested the ability of both cuffs to prevent air leak during a staged recruitment manoeuvre. In the radiological study, we recorded the degree of anatomical distortion of the trachea from both cuffs in the antero-posterior (AP) and transverse tracheal diameters. We performed statistical analysis using non-inferiority tests.

**Results:**

In the bench-top study, the LVLP cuff achieved a plateau at a mean height of 25.2 cmH2O (SD 0.34). In contrast, the conventional cuff failed to maintain any water above the cuff and a plateau could not be measured. In the clinical study, the mean pressure at which air leak occurred was 30.0 +/- 0.8 cmH2O (SD 3.8) using the LVLP cuff and 32.4 +/- 0.7 cmH2O (SD 3.0) using the conventional cuff. In the radiological study, the mean degree of anatomical distortion of the trachea in AP and transverse tracheal diameter was 2.9 +/- 2.2 mm (SD 2.1) and 1.8 +/- 1.4 mm (SD 1.4) using the LVLP cuff and 4.4 +/- 1.3 mm (SD 1.4) and 2.6 +/- 1.5 mm (SD 1.6) using the conventional cuff.

**Conclusions:**

The bench-top and clinical studies both demonstrated that the LVLP cuff exerted approximately 30 cmH2O of pressure on the tracheal wall. These results are supported by our radiological study. We conclude that the LVLP cuff exerts an acceptable amount of pressure on the tracheal wall when it is operated at the recommended intracuff pressure.

## Background

It is important to ensure an appropriate amount of pressure is exerted on the tracheal wall by an endotracheal tube cuff for two opposing reasons. Firstly, a higher pressure is desirable in order to form an effective seal, which reduces the pulmonary aspiration [1]. Secondly, a lower pressure is desirable in order to minimise pressure injury to the tracheal wall [2-4]. Consequently, it has been recommended that an endotracheal tube cuff should exert between 20 and 30 cmH2O of pressure on the tracheal wall [2]. This is thought to represent a balance between preventing pulmonary aspiration and protecting the tracheal wall from pressure injury.

Traditional endotracheal tube cuffs are high-volume, low-pressure (HVLP) cuffs. These cuffs should not be fully inflated when used. This has two important consequences. Firstly, there are longitudinal folds in cuff wall because the cuff is not under tension. Secondly, the pressure exerted on the tracheal wall by the cuff is equal to the intracuff pressure. At an intracuff pressure of 30 cmH2O, the HVLP cuff exerts approximately 30 cmH2O of pressure on the tracheal wall. Therefore, it is recommended that HVLP cuffs are operated at an intracuff pressure of 30 cmH2O.

In clinical practice, HVLP cuffs have been shown to allow pulmonary aspiration at an intracuff pressure of 30 cmH2O [5,6]. This occurs along the longitudinal folds, which develop in the cuff wall. It also appears that HVLP cuffs are routinely overinflated. Recent studies using HVLP cuffs have reported mean intracuff pressures between 35-62 cmH2O [7-9]. The desire to minimise pulmonary aspiration could explain why HVLP cuffs are routinely overinflated.

The Lotrach endotracheal tube has a unique low-volume, low-pressure (LVLP) cuff, which has been designed to prevent pulmonary aspiration and avoid pressure injury to the tracheal wall [10]. The cuff is designed to be fully inflated when used. This has two important consequences. Firstly, the cuff does not develop longitudinal folds in cuff wall because the cuff is under tension. Secondly, the pressure exerted on the tracheal wall by the cuff is equal to the intracuff pressure minus the sum of elastic forces within the cuff. The sum of the elastic forces within the cuff is approximately 50 cmH2O. At an intracuff pressure of 80 cmH2O, the LVLP cuff exerts approximately 30 cmH2O of pressure on the tracheal wall. Therefore, it is recommended that the LVLP cuff is operated at an intracuff pressure of 80 cmH2O. The Lotrach also incorporates a cuff pressure controller to maintain an optimal intracuff pressure over time. The cuff has already been shown to prevent pulmonary aspiration in a pig model, in anaesthetised patients and in the critically ill [11]. The aims of this study were to estimate the pressure exerted on the tracheal wall by a HVLP cuff and the LVLP cuff in a bench-top, clinical and radiological study.

## Method

This prospective, observational study was conducted at the Queen Elizabeth Hospital Intensive Care Unit, Norfolk, UK. We used the Softseal [HVLP, Polyvinylchloride, internal diameter 8 mm, Portex, UK] endotracheal tube and the Lotrach [LVLP, Silicone, internal diameter 8 mm, Venner Medical, Singapore] endotracheal tube. All equipment was used according to the manufacturers' instructions.

### Bench-top study

We placed the HVLP cuff in a rigid cylindrical tube with an internal diameter of 22 mm as an approximate model of normal adult human trachea and used a constant pressure device [Tracoe cuff pressure controller, Tracoe, Frankfurt, Germany] to inflate the HVLP cuff [12]. The HVLP cuff was overpressurised to an intracuff pressure of 50 cmH2O while a column of water was instilled above the cuff to a height of 40 cm. At an intracuff pressure of 50 cmH2O, the HVLP cuff exerts approximately 50 cmH2O of pressure on the tracheal wall. Next, the intracuff pressure was reduced to 30 cmH2O. At an intracuff pressure of 30 cmH2O, the HVLP cuff exerts approximately 30 cmH2O of pressure on the tracheal wall. The height of the column of water was re-measured once column of water had reached a plateau.

Theoretically, the column of water would fall until it reached a height equal to the pressure exerted by the cuff on the tracheal wall, provided that the cuff did not continue to leak. If the cuff did continue to leak, it was assumed that the cuff was not fully inflated and the leakage occurred along the longitudinal folds in the cuff wall. In this instance, the intracuff pressure was assumed to be equal to the pressure exerted on the tracheal wall. The experiment was repeated 10 times so that any variation was averaged out. We did not attempt to assess amount or speed of fluid leakage past the cuff except to identify the pressure at which leakage stopped.

The same protocol was used with the LVLP cuff. The LVLP cuff was overpressurised to an intracuff pressure of 100 cmH2O while a column of water was instilled above the cuff to a height of 40 cm. At an intracuff pressure of 100 cmH2O, the LVLP cuff exerts approximately 50 cmH2O of pressure on the tracheal wall. Next, the intracuff pressure was reduced to 80 cmH2O. At an intracuff pressure of 80 cmH2O, the LVLP cuff exerts approximately 30 cmH2O of pressure on the tracheal wall. The height of the column of water was re-measured once column of water reached a plateau. The experiment was repeated 10 times so that any variation was averaged out.

### Clinical study

We use positive end expiratory pressure (PEEP) during recruitment manoeuvres in critically ill patients. We hypothesised that there would be gas leakage past the endotracheal tube cuff during a recruitment manoeuvre once the PEEP exceeded the pressure exerted on the tracheal wall by the cuff. All patients who required intubation over a 6 month period were included in our analysis. Patients were allocated to each group depending on their anticipated duration of intubation. Those patients who were anticipated to require more than 24 hours of intubation were intubated with a LVLP cuff. Forty eight patients were intubated with the HVLP cuff and 54 patients with the LVLP cuff.

Both cuffs were operated at the recommended intracuff pressure: the HVLP cuff at 30 cmH2O and the LVLP cuff to 80 cmH2O. At these intracuff pressures, both cuffs exert approximately 30 cmH2O of pressure on the tracheal wall. Each patient underwent a staged recruitment manoeuvre while the intracuff pressure was maintained using a constant pressure device [Tracoe cuff pressure control, Tracoe, Frankfurt, Germany]. The PEEP was set to 15 cmH2O and then increased in 5 cmH2O increments every 5 seconds until 40 cmH2O was achieved. A second observer auscultated the anterior neck and the pressure at which air leak was heard was recorded. In practice, the point of air leakage was obvious and heard at the bedside along with the observation of the brisk bubbling of gas escape at the mouth.

Theoretically, there would be no air leak up to a PEEP of 30 cmH2O. Above this, the PEEP would exceed the pressure exerted on the tracheal wall by the cuff and an air leak heard. Statistical analysis was performed using a two sided 95% confidence interval non-inferiority test. We hypothesised that the mean difference in the pressure exerted on the tracheal wall by the HVLP cuff and the LVLP cuff was within a clinically relevant magnitude. A figure 20% either side of 30 cmH2O was chosen to delineate the limits of a clinically relevant magnitude [13].

### Radiological study

We hypothesised that the anatomical distortion of the trachea caused by the inflation of the cuffs would be similar if the pressure exerted on the tracheal wall from both cuffs was similar. Both cuffs were operated at the recommended intracuff pressure: the HVLP cuff at 30 cmH2O and the LVLP cuff to 80 cmH2O. At these intracuff pressures, both cuffs exert approximately 30 cmH2O of pressure on the tracheal wall.

All patients who required chest CT examinations as part of their ongoing medical care over a 6 month period were included in our analysis. In this part of the study, the intracuff pressure of the HVLP cuff was not controlled. In contrast, the intracuff pressure in the LVLP cuff was controlled because the device incorporates a cuff pressure controller. An independent radiologist examined the images of seven sequential patients who were intubated using the HVLP cuff and six sequential patients who were intubated using the LVLP cuff.

**Figure 1:**
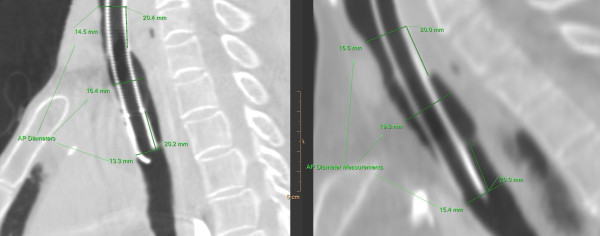
**Examples of the CT images in a sagittal plane of the LVLP (left) and HVLP cuff (right) *in situ*, delineating the mid cuff level, 20 mm above and 20 mm below cuff measurements**.

The tracheal antero-posterior (AP) and transverse diameters were measured at the mid-cuff level, and 20 mm above the cuff and 20 mm below the cuff where there was no contact with the trachea (Figure 1). The degree of anatomical distortion of the trachea was recorded. Anatomical distortion was defined as the difference in diameter between the mid-cuff and the mean of the above and below cuff diameters. Statistical analysis was performed using a two sided 95% confidence interval non-inferiority test. We hypothesised that the mean difference in anatomical distortion of the trachea by the HVLP cuff and the LVLP cuff was within a clinically relevant magnitude. A figure 20% either side of the mean of the above and below cuff diameters was chosen to delineate the limits of a clinically relevant magnitude [13].

### Ethical considerations

This study was conducted with prospective approval from the Local Research and Ethics Committee. The requirement for patient consent or next of kin consent was waived because the patients were unconscious, the data was observational data from our routine practice, the data was collected for audit, service surveillance and improvement purposes and the data was anonymised. In the clinical study, each patient underwent a recruitment manoeuvre as part of his or her routine respiratory care. The data from this practice was recorded in the patients' electronic medical record. In the radiological study, each patient underwent a chest CT examination as part of as part of his or her medical care. The data from this practice was recorded in the patients' electronic medical record.

## Results

### Bench top study

The HVLP cuff failed to maintain any water above the cuff and a plateau could not be measured. Therefore, the pressure exerted on the tracheal wall was assumed to be equal to the intracuff pressure. The LVLP cuff achieved a plateau at a mean height of 25.2 cmH2O (SD 0.34). There was no leakage of water past the cuff once the column of water had reached a plateau. No statistical analysis was performed because the HVLP cuff did not achieve a plateau.

### Clinical study

The mean pressure at which air leak occurred using the HVLP cuff was 32.4 +/- 0.7 cmH2O (SD 3.0). The mean pressure at which air leak occurred using the LVLP cuff was 30.0 +/- 0.8 cmH2O (SD 3.8). There was a statistically significant difference between the two groups: the 95% confidence intervals at which air leak occurred did not overlap. However, the limits of our clinically relevant magnitude were 24 cm and 36 cm. The confidence intervals for each cuff were enclosed within these limits and so difference was deemed not clinically relevant.

### Radiological study

The mean duration of time between intubation and chest CT examination was 2.6 hours (SD 4.4) in the HVLP cuff group and 192.4 hours (SD 228.9) in the LVLP cuff group. The tracheal AP and transverse diameters at the mid-cuff level, and 20 mm above the cuff and 20 mm below the cuff are shown for the HVLP cuff and the LVLP cuff in tables 1 and 2. The mean degree of anatomical distortion of the trachea in AP and transverse tracheal diameter using the HVLP cuff was 4.4 +/- 1.3 mm (SD 1.4) and 2.6 +/- 1.5 mm (SD 1.6) and using the LVLP cuff was 2.9 +/- 2.2 mm (SD 2.1) and 1.8 +/- 1.4 mm (SD 1.4). This represented a percentage change from baseline diameter of the trachea in AP and transverse tracheal diameter of 19.2% and 13.0% using the HVLP cuff and 14.1% and 9.8% using the LVLP cuff. There was not a statistically significant difference between the two groups: the 95% confidence intervals did overlap. However, the limits of our clinically relevant magnitude were 18.15 +/- 3.6 mm for the HVLP cuff and 16.9 +/- 3.4 mm for the LVLP cuff. The confidence intervals for the HVLP cuff exceeded these limits in both diameters and the LVLP cuff in the AP diameter and so the degree of anatomical distortion was deemed clinically relevant.

**Table 1:** The tracheal AP and transverse diameters at 20 mm above the cuff, the mid-cuff level and 20 mm below the cuff are shown for the HVLP.

Level	Diameter above cuff (mm)		Diameter at mid cuff (mm)		Diameter below cuff (mm)	
	**AP**	**Transverse**	**AP**	**Transverse**	**AP**	**Transverse**

1	14	17	22	20	21	14

2	25	27	27	22	17	19

3	24	15	25	24	18	25

4	16	15	17	16	14	15

5	17	16	21	20	13	16

6	24	21	26	21	20	23

7	17	15	20	18	15	15

Mean (SD)	19.6 (4.6)	18 (4.5)	22.6 (3.6)	20.1 (2.6)	16.9 (3.0)	18.1 (4.3)

**Table 2:** The tracheal AP and transverse diameters at 20 mm above the cuff, the mid-cuff level and 20 mm below the cuff are shown for the LVLP.

Level	Diameter above cuff (mm)		Diameter at mid cuff (mm)		Diameter below cuff (mm)	
	**AP**	**Transverse**	**AP**	**Transverse**	**AP**	**Transverse**

1	15	16	16	16	13	14

2	21	19	21	20	14	19

3	18	19	19	17	18	17

4	16	16	17	18	17	17

5	20	21	20	19	19	17

6	17	16	25	21	13	13

Mean (SD)	17.8 (2.3)	17.8 (2.1)	19.7 (3.2)	18.5 (1.9)	15.7 (2.7)	16.2 (2.2)

## Discussion

It has been recommended that the pressure exerted on the tracheal wall by an endotracheal tube cuff should be between 20 and 30 cmH2O [2]. This is thought to represent a balance between preventing pulmonary aspiration and protecting the trachea from pressure injury. Previously, the LVLP cuff has been shown to prevent pulmonary aspiration in a pig model, in anaesthetised patients and in the critically ill [11]. This is the first study to investigate the pressure exerted by the LVLP cuff on the tracheal wall *in vitro *and *in vivo*.

Our bench top study estimated that the LVLP cuff transmitted approximately 30 cmH2O of pressure on the tracheal wall. This is demonstrated by the fact that there was only leakage of water past the cuff until the water column reached a height of 25.2 cm. This differs from 30 cmH2O because there is a range of acceptable values, which are allowed as part of the quality control during cuff manufacture. All LVLP cuffs operate within the range of 25-35 cmH2O. In contrast to the HVLP cuff, the LVLP cuff provided an effective seal at this pressure. The clinical study also estimated that the LVLP cuff exerted approximately 30 cmH2O of pressure on the tracheal wall, which was similar to that exerted by the HVLP cuff. These results are supported by our radiological study, which found that the degree of anatomical distortion of the trachea was similar in patients using the HVLP and LVLP cuff in both the AP and transverse diameters in normal clinical practice. Put together, these results suggest that the LVLP cuff exerts an acceptable amount of pressure on the tracheal wall, despite it having an intracuff pressure of 80 cmH2O.

In the radiological study, the mean duration of time between intubation and chest CT examination was substantially longer in patients with LVLP cuffs (192.4 hours, SD 228.9) compared to patients with HVLP cuffs (2.6 hours, SD 4.4). This difference occurred because those patients who were anticipated to require more than 24 hours of intubation were intubated with a LVLP cuff and many of these patients went on to require a substantially longer period of intubation. Anatomical distortion of the trachea is known to occur after prolonged intubation [14]. Consequently, there is a bias in our study that favours anatomical distortion in patients with a LVLP cuff because they were intubated for a longer period time. This data further supports our assertion that the LVLP cuff exerts an acceptable amount of pressure on the tracheal wall when it is operated at the recommended intracuff pressure.

The finding that the degree of anatomical distortion was deemed clinically relevant only in patients with HVLP cuffs was unexpected. A possible explanation is that the baseline tracheal diameters (as judged by the above and below cuff measurements) were lower in patients with HVLP cuffs. Therefore, the difference in the degree of the anatomical distortion with the HVLP cuffs can be attributed to the range of the patients' tracheal diameters and the geometrical configuration of the HVLP cuff. Interestingly, the degree of anatomical distortion was different in the AP and transverse tracheal diameters for both cuffs, which suggests that either the pressure from a cuff is not exerted homogenously on the trachea or that the compliance of the trachea is different in the two different axes.

Previously, the pressure exerted on the tracheal wall by endotracheal tube cuffs has been estimated using calculations from balloons interposed between cuff and trachea, implantable transducers cuff, gas flowing across the cuff-trachea contact area through hollow sleeves and compliance curves [15-18]. There are common methodological problems with each technique, which may be categorised under the headings measuring probe error, pressure measurement volume displacement error and volume aliquot inflation error.

Measuring probe error: interposing a transducer between the cuff and the trachea produces error because the transducer distorts the geometry of the trachea and/or the cuff [16]. In addition, if the transducer is fractionally on the luminal side of the trachea, the pressure may be overestimated. Alternatively, if the transducer is fractionally recessed into the trachea, the pressure may be underestimated. Furthermore, the position of the transducer in the trachea varies throughout inflation because the trachea is distensible while the transducer is not. Pressure measurement volume displacement error: gas within the measuring system produces error because it is compressible [19]. The greater the volume or pressure of the gas in the measuring system produces a greater underestimate of the true pressure. Volume aliquot inflation error: the addition of fixed aliquots of volume produces error because different cuffs have different compliance characteristics [17]. A small increase in volume for a cuff with low compliance will produce a greater increase in pressure relative to a cuff with high compliance.

In this study, we estimated the pressure exerted on the tracheal wall by the cuff by measuring the ability of the cuff to support a column of water *in vitro *and a column of gas *in vivo*. Advantages of these techniques are that there is no transducer distorting the trachea/cuff, the measuring system does not contain compressible gas and the measurement is not affected by the compliance characteristics of the cuff itself. A disadvantage of these techniques is that measurement is not possible if there is a leak in the system as exemplified by our bench top study.

A limitation of the radiological study was that it was observational data. This meant the intracuff pressure in patients with HVLP cuffs was not controlled. In contrast, the intracuff pressure in patients with LVLP cuffs was controlled because the device incorporates a cuff pressure controller. Therefore, any conclusions that are drawn between the two groups of patients are limited by the lack of control of the intracuff pressure in patients with HVLP cuffs. However, our results do reflect the usage of the two devices in normal clinical practice. The LVLP cuff is always used with a cuff pressure controller and so will always benefit from a second to second intracuff pressure adjustment back to normal. The HVLP cuffs commonly do not have a cuff pressure control system, notable exceptions being the Tracoe device described above and the Lanz endotracheal tube (Covidien, Mansfield, MA, USA). Furthermore, there were a limited number of patients in our radiological study because only patients who required chest CT examinations as part of their medical care were included in our analysis. However, these represented all of the available patients at the time of our analysis. Another limitation is that our study does not address the effect of the mucosal injury caused by shear forces on the trachea, which may occur with movement in a longitudinal or rotational basis between cuff and tracheal wall. This will require a further study.

## Conclusions

The bench-top study estimated that the LVLP cuff exerted approximately 30 cmH2O of pressure on the tracheal wall. The clinical study also estimated that the LVLP cuff exerted approximately 30 cmH2O of pressure on the tracheal wall, which was similar to that exerted by the HVLP cuff. These results are supported by our radiological study, which found that the degree of anatomical distortion of the trachea was similar using the HVLP and LVLP cuff in normal clinical practice. We conclude that the LVLP cuff exerts an acceptable amount of pressure on the tracheal wall when it is operated at the recommended intracuff pressure of 80 cmH2O.

## Competing interests

Dr Peter Young is the inventor of the Lotrach endotracheal tube. In the past he has received funding and consultancy fees from Venner Medical. This work was not funded or financed by Venner Medical. Dr Peter Young is a minor shareholder in the intellectual property ownership of the Lotrach endotracheal tube and tracheal seal monitor (cuff pressure controller).

The remaining authors do not declare any conflict of interest or competing interest.

## Authors' contributions

AD carried out the bench-top and radiological study, drafted the write up for the bench-top study and is the lead author of the submitted manuscript. RS carried out the clinical study, drafted the write up for this section and gave final approval for the version to be published. MC interpreted the CT scans in the radiology study, drafted the write up for this section and gave final approval for the version to be published. MB devised the bench-top study, critically revised the manuscript and gave final approval for the version to be published. PY devised the clinical and radiological studies, critically revised the manuscript and gave final approval for the version to be published.

## Pre-publication history

The pre-publication history for this paper can be accessed here:

http://www.biomedcentral.com/1471-2253/10/21/prepub

## References

[B1] Metheny NA Schallom L Oliver DA Clouse RE Gastric residual volume and aspiration in critically ill patients receiving gastric feedings Am J Crit Care 2008 17 6 512 519 18978236 PMC2627559

[B2] Seegobin RD van Hasselt GL Endotracheal cuff pressure and tracheal mucosal blood flow: endoscopic study of effects of four large volume cuffs Br Med J 1984 288 6422 965 968 10.1136/bmj.288.6422.965 PMC1442489 6423162

[B3] Poetker DM Ettema SL Blumin JH Toohill RJ Merati AL Association of airway abnormalities and risk factors in 37 subglottic stenosis patients Otolaryngol Head Neck Surg 2006 135 3 434 437 10.1016/j.otohns.2006.04.013 16949978

[B4] Zias N Chroneou A Tabba MK Gonzalez AV Gray AW Lamb CR Riker DR Beamis JF Post tracheostomy and post intubation tracheal stenosis: report of 31 cases and review of the literature BMC Pulm Med 2008 8 18 10.1186/1471-2466-8-18 18803874 PMC2556644

[B5] Young PJ Rollinson M Downward G Henderson S Leakage of fluid past the tracheal tube cuff in a benchtop model Br J Anaesth 1997 78 5 557 562 9175972 10.1093/bja/78.5.557

[B6] Seegobin RD van Hasselt GL Aspiration beyond endotracheal cuffs Can Anaesth Soc J 1986 33 273 279 10.1007/BF03010737 3719428

[B7] Sengupta P Sessler DI Maglinger P Wells S Vogt A Durrani J Wadhwa A Endotracheal tube cuff pressure in three hospitals, and the volume required to produce an appropriate cuff pressure BMC Anesthesiol 2004 4 1 8 10.1186/1471-2253-4-8 15569386 PMC535565

[B8] Sathishkumar S Young P Tracheal cuff pressure - a survey of clinical practice Br J Anaesth 2002 88 3 456 11990290

[B9] Chopra M Jones L Boulanger C Benger J Higginson I Williamson D Younge P Lloyd G Prospective observational measurement of tracheal tube cuff pressures in the emergency department Emerg Med J 2010 27 4 270 271 10.1136/emj.2009.075200 20385676

[B10] Fletcher AJ Ruffell AJ Young PJ The LoTrach system: its role in the prevention of ventilator-associated pneumonia Nurs Crit Care 2008 13 5 260 268 10.1111/j.1478-5153.2008.00286.x 18816312

[B11] Young PJ Pakeerathan S Blunt MC Subramanya S A low-volume, low-pressure tracheal tube cuff reduces pulmonary aspiration Crit Care Med 2006 34 3 632 639 10.1097/01.CCM.0000201406.57821.5B 16505646

[B12] Chandler M Crawley BE Rationalization of the selection of tracheal tubes Br J Anaesth 1986 58 1 111 116 10.1093/bja/58.1.111 3942660

[B13] Riffenburgh RH Equivalence testing Statistics in Medicine 2006 2 London: Elsevier 386 396

[B14] Sarper A Ayten A Eser I Ozbudak O Demircan A Tracheal stenosis after tracheostomy or intubation: review with special regard to cause and management Tex Heart Inst J 2005 32 2 154 158 16107105 PMC1163461

[B15] Leigh JM Maynard JP Pressure on the tracheal mucosa from cuffed tubes Br Med J 1979 1 6172 1173 1174 10.1136/bmj.1.6172.1173 444996 PMC1599329

[B16] Dobrin P Canfield T Cuffed endotracheal tubes: mucosal pressures and tracheal wall blood flow Am J Surg 1977 133 5 562 568 10.1016/0002-9610(77)90008-3 860779

[B17] Knowlson GT Bassett HF The pressures exerted on the trachea by endotracheal inflatable cuffs Br J Anaesth 1970 42 10 834 837 10.1093/bja/42.10.834 4920121

[B18] MacKenzie CF Klose S Browne DR A study of inflatable cuffs on endotracheal tubes. Pressures exerted on the trachea Br J Anaesth 1976 48 2 105 110 10.1093/bja/48.2.105 766796

[B19] Cox PM Schatz ME Respiratory therapy. Pressure measurements in endotracheal cuffs: a common error Chest 1974 65 1 84 87 10.1378/chest.65.1.84 4809338

